# A practical solution to estimate the sample size required for clinical prediction models generated from observational research on data

**DOI:** 10.1186/s41747-022-00276-y

**Published:** 2022-06-01

**Authors:** Carlos Baeza-Delgado, Leonor Cerdá Alberich, José Miguel Carot-Sierra, Diana Veiga-Canuto, Blanca Martínez de las Heras, Ben Raza, Luis Martí-Bonmatí

**Affiliations:** 1 Biomedical Imaging Research Group (GIBI230-PREBI) at La Fe Health Research Institute and the Imaging La Fe node of the Distributed Network for Biomedical Imaging (ReDIB) Unique Scientific and Technical Infrastructures (ICTS), Valencia, Spain; 2 Department of Applied Statistics, Operations Research and Quality, Universitat Politècnica de València, Valencia, Spain; 3 Radiology Department, Hospital Universitario y Politécnico La Fe, Valencia, Spain; 4 Pediatric Oncology Department, Hospital Universitario y Politécnico La Fe, Valencia, Spain

**Keywords:** Sample size calculation, Clinical predictive models, PRIMAGE, Paediatric oncology, Radiology

## Abstract

**Background:**

Estimating the required sample size is crucial when developing and validating clinical prediction models. However, there is no consensus about how to determine the sample size in such a setting. Here, the goal was to compare available methods to define a practical solution to sample size estimation for clinical predictive models, as applied to Horizon 2020 PRIMAGE as a case study.

**Methods:**

Three different methods (Riley’s; “rule of thumb” with 10 and 5 events per predictor) were employed to calculate the sample size required to develop predictive models to analyse the variation in sample size as a function of different parameters. Subsequently, the sample size for model validation was also estimated.

**Results:**

To develop reliable predictive models, 1397 neuroblastoma patients are required, 1060 high-risk neuroblastoma patients and 1345 diffuse intrinsic pontine glioma (DIPG) patients. This sample size can be lowered by reducing the number of variables included in the model, by including direct measures of the outcome to be predicted and/or by increasing the follow-up period. For model validation, the estimated sample size resulted to be 326 patients for neuroblastoma, 246 for high-risk neuroblastoma, and 592 for DIPG.

**Conclusions:**

Given the variability of the different sample sizes obtained, we recommend using methods based on epidemiological data and the nature of the results, as the results are tailored to the specific clinical problem. In addition, sample size can be reduced by lowering the number of parameter predictors, by including direct measures of the outcome of interest.

## Key points

- Estimating the appropriate sample size in clinical prediction model development is mandatory to guarantee the robustness of the results.
- The selected method is designed to be applied to epidemiological data and based on the nature of outcomes.
- Strategies based on the selection and reduction of predictor variables are proposed to reduce sample size.
- The expected recruitment in PRIMAGE project fits the estimated sample size.

## Background

In research studies, including experimental clinical trials and observational studies, estimating the sample size is essential to ensure that the results will be conclusive and representative of the studied cohort [1]. Inappropriate size estimates generate uncertainties to provide reliable and reproducible answers to the questions the study intends to address [2]. Lower number of cases limits the capacity to detect existing differences, whereas larger sample size provides reliable results at the cost of increasing resources, expenses and the duration of the study [3].

Classic univariate research questions involve both descriptive (estimates of a population parameter or change) and analytical (association and correlation studies) statistics. Both methods apply a collection of well-described equations that enable the direct estimation of the needed sample size [4]. For these assessments, a prior estimate of the parameter to be studied and of its confidence interval, or the effect size and both the acceptable type I and type II errors, are needed to perform the calculations [3]. The sample size estimation in clinical predictive models extracted from observational data is more complex, since suitable direct equations are not readily available [5, 6].

**Fig. 1:**
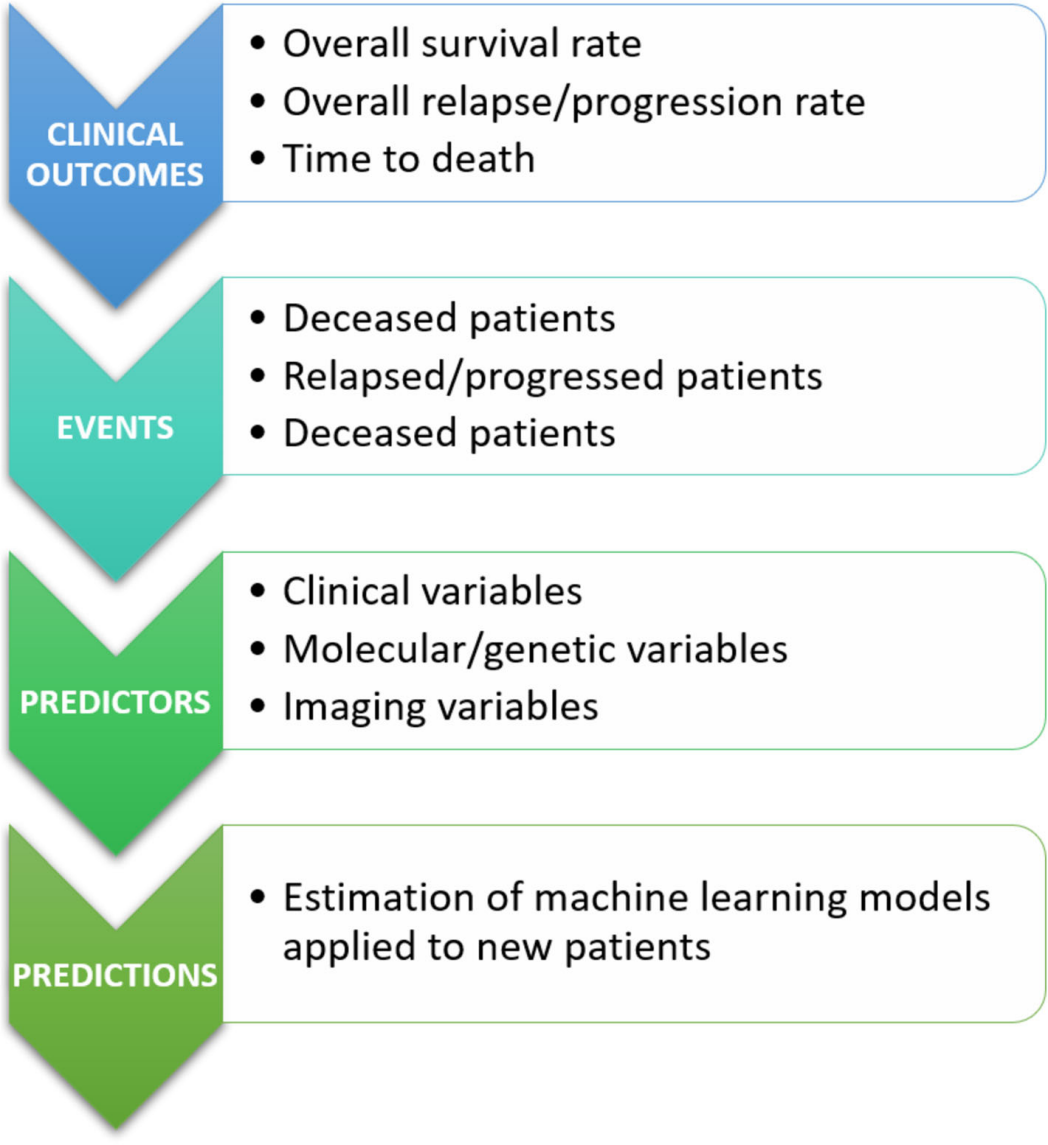
Factors involved in sample size estimation and model development. Example of the clinical outcomes, events, predictors and predictions applied to neuroblastoma

When developing predictive models, a widely used “rule of thumb” to estimate sample size is that based on simulation studies conducted in the 1990s, stating that at least 10 events per predictor variable (EPP) must be included [7–9]. It should be noted that in these observational predictions, events refer to the number of patients in the sample with the clinical characteristic of interest (Fig. 1). Nevertheless, this rule has been widely questioned due to the context-specific nature of the EPP required, which may correspond to a number other than 10 EPP [10, 11].

Two methodologies that go beyond simple rule of thumb are considered as a baseline for sample size estimation in this study. For logistic and Cox regression models, the 10 EPP rule of thumb can be relaxed to as low as to 5 EPP depending on the nature of the model, either logistic or Cox regression, and whether the primary predictor variable is binary or continuous [12]. A new method to calculate the sample size for parametric predictive models was proposed based on different factors, such as disease prevalence in the population, the number of predictor variables, the number of participants, and the expected fit of the regression model [13]. In this four-step method to calculate the sample size for estimation models, each step claims to meet a different criterion related to model performance. These four calculations vary depending on the type of outcome of the model (binary, continuous or time-to-event) and eventually, the largest sample size obtained is chosen. Finally, model validation has an essential role to demonstrate that an estimative algorithm is reproducible and can be consistently applied in clinical practice. In this case, there is a higher consensus that the minimum sample size for a robust validation should have at least 100 events [14].

This study aimed to use the PRIMAGE project as a use-case to apply and compare the aforementioned methods to estimate the sample size required for both model development and validation. The estimations will be performed to different scenarios regarding the clinical endpoints for neuroblastoma (NB) and diffuse intrinsic pontine glioma (DIPG) patients [15]. Secondly, the required sample size was compared with the expected recruitment within the project, and different approaches were explored to reduce the required sample size.

## Methods

### PRIMAGE project

PRIMAGE (PRedictive In silico Multiscale Analytics to support cancer personalised diaGnosis and prognosis, Empowered by imaging biomarkers) is a Horizon 2020 funded research project (RIA, topic SC1-DTH-07-2018), an in silico observational study for the training and validation of machine learning algorithms and multiscale prediction models [15]. This project aims to offer precise clinical assistance in the most relevant paediatric cancers: NB and DIPG. The data repository contains a high number of variables, including clinical, molecular and genetic data (above 300 different variables), as well as imaging data (more than 100 radiomic features). Throughout the project, machine learning and image processing deep learning algorithms will be used to extract pattern information from the images and link outcome results to known ground-truth diagnosis.

### Sample size estimation

The methodology described by Riley [13, 16, 17] was that chosen to calculate the sample size needed to develop the computational, in silico, observational predictive model to be used in the PRIMAGE project. PRIMAGE aims to generate and validate predictive tools to diagnose and manage malignant childhood NB and DIPG tumours based on their phenotype and aggressiveness.

The sample size and EPP calculations for the different models generated, either binary or time-to-event, were implemented in R using the *pmsampsize* package [13]. For comparison, the sample size was also estimated using the 10 EPP “rule of thumb” and the updated 5 EPP rule. The calculations were applied to different scenarios for both NB and DIPG based on the clinical endpoints of interest described in the project, such as mortality risk at certain timepoint, time to death, time to relapse/progression, relapse/progression risk, event-free survival rate, and progression-free survival (PFS). In the case of NB, some of the clinical endpoints exclusively referred to the high-risk (HR) sub-group, due to their characteristics and clinical interest. A list of all these scenarios can be found in Table 1.

**Table 1:** Clinical endpoints and model type

sTumor type	Description	Type of outcome
Neuroblastoma	5-year mortality risk	Binary
Neuroblastoma	Relapse/progression risk	Binary
Neuroblastoma	Time-to-death	Time-to-event
HR-Neuroblastoma	HR 5-year mortality risk	Binary
HR-Neuroblastoma	HR 5-year relapse/progression risk	Binary
HR-Neuroblastoma	HR-time to relapse/progression	Time-to-event
DIPG	1-year mortality risk	Binary
DIPG	2-year mortality risk	Binary
DIPG	1-year progression risk	Binary
DIPG	Time-to-death	Time-to-event
DIPG	Time-to-progression	Time-to-event

List of the clinical endpoints for neuroblastoma, HR neuroblastoma and DIPG tumors for which the sample size was determined, and the type of outcome for each of these. *DIPG* diffuse intrinsic pontine glioma, *HR* high-risk

### Epidemiological data

The clinical endpoint data required by the *pmsampsize* package [13] to perform the calculations was obtained from previous studies after a detailed review [18–21] (Table 2). In the case of the endpoints for NB, the data for the 5-year OS rate (30.7%) and time-to-death (median time-to-event 24.2 months, time of follow-up 60 months) were obtained from [18], and the data for the prevalence of relapse/progression (25.75%) was from [20]. For HR NB, the data regarding the 5-year OS rate (50%), the 5-year event-free survival rate (40.8%), the prevalence of relapse (56.1%), the median time-to-relapse (19.08 months) and the median follow-up time (72.12 months) were all collected from [19]. For the DIPG endpoints, an extensive systematic review [21] provided all the necessary data for the calculations of the different endpoints, including: the 1-year (45%) and 2-year OS rates (16.9%), the 1-year PFS rate (23.5%), the median time-to-death (11.4 months), the follow-up time for the time-to-death model (24 months), and the median time-to-progression (7.7 months) and follow-up time for the time-to-progression model (12 months).

**Table 2:** Required data for sample size calculations

Model	Prevalence	Median *t*_*event*_	*t*_*follow-up*_	Rate	Time point	Follow-up
NB 5-year mortality risk	0.307	–	–	–	–	–
NB relapse/progression risk	0.258	–	–	–	–	–
NB time to death	0.307	24.2	60	0.0063	24	24
HR-NB 5-year mortality risk	0.500	–	–	–	–	–
HR-NB 5-year relapse/progression risk	0.408	-	–	–	–	–
HR-NB time to relapse/progression	0.561	19.08	72.12	0.0132	24	24
DIPG 1-year mortality risk	0.450	–	–	–	–	–
DIPG 2-year mortality risk	0.169	–	–	–	–	–
DIPG 1-year progression risk	0.235	–	–	–	–	–
DIPG time to death	0.169	11.4	24	0.0077	24	24
DIPG time to progression	0.235	7.7	12	0.0214	12	12

Data related to each clinical endpoint for which the sample size has been determined. The number of parameters and the value of *R* is the same for all 11 scenarios (30 and 0.3, respectively) and thus, only the prevalence is required for the binary models

### pmsampsize settings

Among the different parameters of *pmsampsize*, shrinkage (that is the regularisation of the variability in the model’s predictions to reduce overfitting) was set to the default value of 0.9 and the number of predictor variables was initially fixed to 30. This initial value was chosen as a conservative one, since in clinical predictive models including radiomic features, the number of predictor variables is usually lower, between 2 and 20 [22–26]. Moreover, the risk of overfitting or spurious discoveries would increase with the number of predictor parameter included in the models [27, 28]. Regarding the expected fit of the model, the Cox-Snell *pseudo R*^2^ (*R*^2^_CS_) required by the equations ranged from 0 to a max(*R*^2^_CS_) < 1, depending on the prevalence of the outcome. To normalize the value of *R*^2^_CS_ in order to compare between different models, Nagelkerke defined another *pseudo R*^2^ (*R*^2^_Nagelkerke_) [29], calculated as the ratio between *R*^2^_CS_ and max(*R*^2^_CS_) (Eq. 1), such that the *R*^2^_CS_ needed by the equations can be obtained from the *R*^2^_Nagelkerke_. With respect to the *R*^2^_Nagelkerke_ value, the authors suggest that in the absence of other information sample sizes should be derived assuming the *R*^2^_CS_ value corresponds to an *R*^2^_Nagelkerke_ of 0.15. However, if the predictor variable includes direct measurements or direct measures of the processes involved in the outcome, they suggest a more appropriate *R*^2^_Nagelkerke_ value of 0.5 [13]. Given that we do have some information on the processes involved but we do not have direct measures, we decided to compromise and chose a *R*^2^_Nagelkerke_ value of 0.3.

For the time-to-event models, the time point of interest for the prediction and the expected average follow-up time for individuals in the dataset used to develop the model was set by experienced paediatric oncologists: 24 months for the time-to-death model of NB, for the time-to-relapse/progression model of HR NB and for the DIPG time-to-death models; and 12 months for the DIPG time-to-progression model (Eq. 1: estimation of the rate of incidence (person-time)).

1 $$ {R}_{Nage/ kerke}^2=\frac{R_{Cox- Snell}^2}{\mathit{\max}\left({R}_{Cox- Snell}^2\right)} $$

One of the parameters required by *pmsampsize* functions to calculate the sample size for time-to-event models is the rate of incidence or person-time rate. Briefly, the rate of incidence is the number of new events during the study follow-up, considered as those patients that present the outcome under study, relative to the total time contributed by all subjects during the observation period (Eq. 2).

2 $$ person- time\ rate=\frac{new\  events\ during\ the\ study\ follow\hbox{-} up}{total\ follow\hbox{-} up\  time} $$

Since this data is difficult to find from previous studies, the person-time rate was estimated following the approach shown in Eqs. 3, 4, and 5 as a function of prevalence, median time-to-event and median follow-up-time. Considering that the median time-to-event is the time at which 50% of subjects become events, the sum of the total time contributed was considered as the number of events multiplied by the median time-to-event (*t*_event_) plus the number of non-events multiplied by the median time of follow-up (*t*_follow-up_: Eqs. 2 and 3).

3 $$ person- time\ rate=\frac{ event s}{ event s\ast median\ {t}_{event}+ events\ast median\ {t}_{follow- up}} $$

Due to the characteristics of the study, only the number of events and the median follow-up time could be found. As a consequence, we used Eq. 4 as an approximation to the incidence rate (person-time), where p is the proportion between the number of events (number of patients in the sample with the clinical characteristics of interest) and the total number of patients in the study (*N*), as stated in Eq. 5.

4 $$ person- time\ rate=\frac{p}{p\ast \left( median\ {t}_{event}\right)+\left(1-p\right)\ast \left( median\ {t}_{follow- up}\right)} $$

5 $$ p=\frac{events}{N} $$

In the cases where it is not possible to identify the median follow-up time but the prevalence for a certain time is available, the time point for which the prevalence data is given may be considered as the follow-up time for non-events, as if all non-events had been censored at that point.

### Sample size variability

The effect of the number of predictor variables on the sample size was studied by executing the *pmsampsize* functions, varying the number of variables from 5 to 30 at intervals of 5, and leaving constant all other conditions of the equations. The variability in sample size as a function of the *R*^2^_Nagelkerke_ was assessed by establishing a value of 0.15 and 0.5 as indicated previously, and to 0.8 in accordance with a hypothetical situation in which the expected fit of the model would be higher. For the time-to-event models, the effect of the ratio between the time point of prediction and the expected time of follow-up was analysed by varying this ratio, such that the higher the ratio the longer the follow-up time relative to the time point, with ratio values between 1 and 4.

### Sample size for model validation

The sample size for model validation was calculated from equation 4, using 100 as a minimum and 200 as a desirable number of events [14].

## Results

### Sample size determination

The sample size for each different endpoint was determined by applying the *pmsampsize* algorithms to data described in Methods. Accordingly, the sample size needed to develop robust clinical predictive models ranged from 1111 to 1397 NB patients, from 1043 to 1060 HR NB patients, and from 1043 to 1345 DIPG patients (Table 3). When more than one endpoint prediction was under study, and therefore more than one sample size was required, the largest estimated sample size should be chosen, such that the definite sample size was selected as the upper limit of the different ranges: 1397 for NB, 1060 for HR NB, and 1345 for DIPG tumours.

**Table 3:** Results of sample size calculations

Model	Riley’s sample size	10 EPP	5 EPP	Riley’s EPP
NB 5-year mortality risk	1111	978	490	11.37
NB relapse/progression risk	1168	1166	584	10.03
NB time to death	1397	978	490	7.00
HR-NB 5-year mortality risk	1043	600	300	17.38
HR-NB 5-year relapse/progression risk	1060	736	369	14.42
HR-NB time to relapse/progression	1060	536	268	11.23
DIPG 1-year mortality risk	1043	668	334	15.65
DIPG 2-year mortality risk	1345	1776	889	7.58
DIPG 1-year progression risk	1208	1278	639	9.46
DIPG time to death	1273	1776	889	7.87
DIPG time to progression	1130	1278	639	9.67

Sample size estimated by Riley’s methodology, the 10 EPP “rule of thumb” and 5 EPP. The number of events per predictor (EPP) variable derived from the sample size obtained with Riley’s methodology was also calculated. *EPP* event per predictor parameter

In order to compare the sample size obtained with other accepted methodologies, sample sizes were also calculated following the 10 EPP “rule of thumb” [7–9] and the 5 EPP estimation [12]. In the first case the sample sizes obtained were smaller, ranging from 978−1166 for the neuroblastoma, 536–736 for the HR neuroblastoma, and 668−1776 for the DIPG models. With the 5 EPP estimations the sample sizes were half those calculated with the 10 EPP rule, 490–584 for neuroblastoma, 268–369 for HR neuroblastoma, and 334–889 for DIPG. Finally, the EPP for the sample size estimated using Riley’s methodology was also obtained from *pmsampsize*, and the number of events per variable were > 10 in more than half of the scenarios analysed and ≥ 7 in the rest of cases.

### Sample size variability

To address the possibility of reducing the sample size while maintaining statistical power, additional calculations were performed in which conditions of the *pmsampsize* equations were varied.

**Fig. 2:**
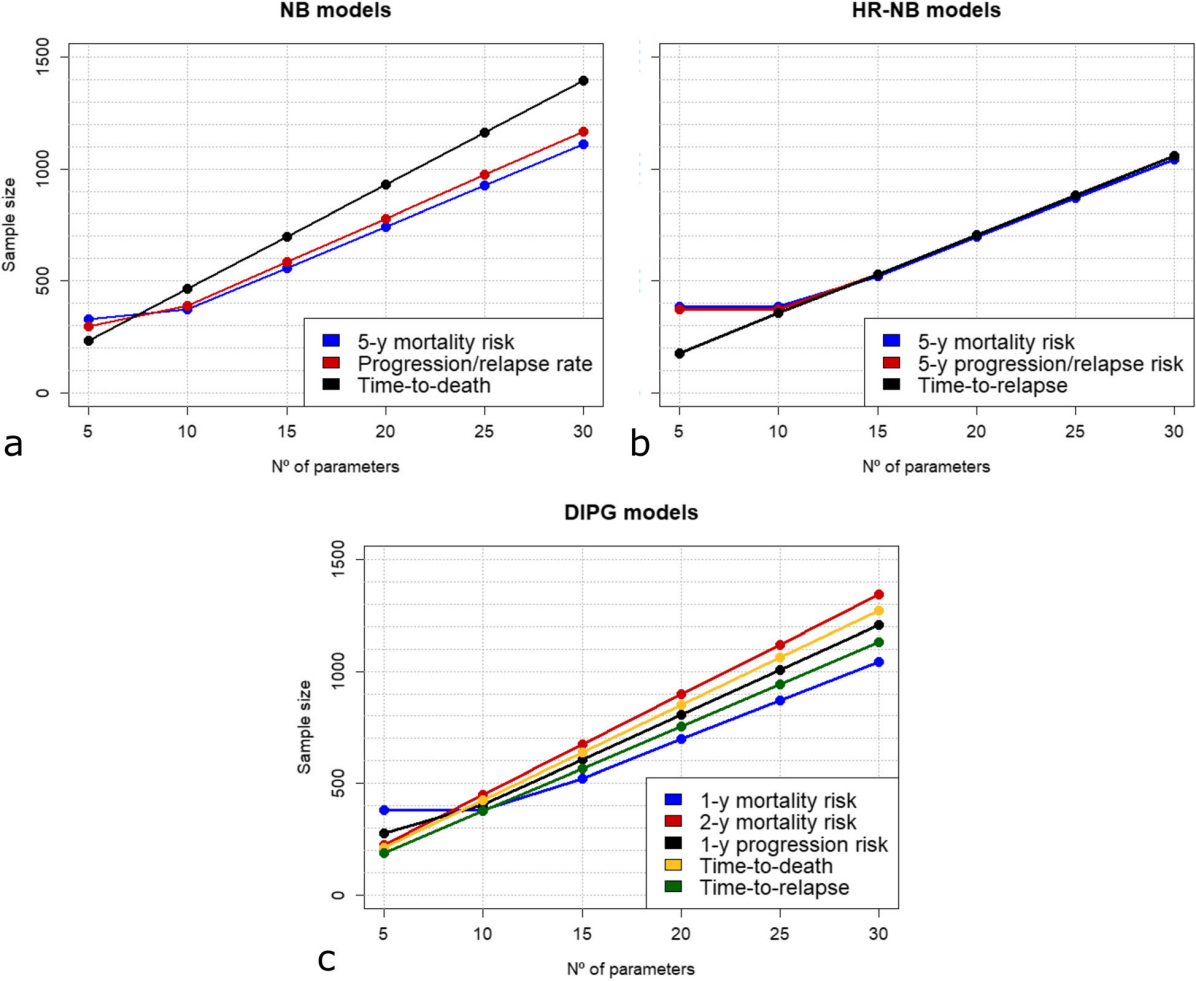
Impact of the number of predictor variables on the sample size. Variability in the sample size relative to the number of predictor variables included in model development for neuroblastoma (**A**), HR neuroblastoma (**B**) and DIPG (**C**). *DIPG* diffuse intrinsic pontine glioma, *HR* high-risk, *NB* neuroblastoma

The variation in the number of predictor variables showed a direct proportional behaviour between sample size and number of variables in the range 10–30 predictor variables for all the 11 scenarios analysed, as well as for 6 scenarios in the 5−30 range (Fig. 2). For example, the sample size can be reduced by half in all 11 scenarios analysed if the number of variables is reduced to a half, from 30 to 15 predictors: 556, 584, and 699 patients in the 5-year mortality risk, relapse risk and time-to-death models for NB; 522, 530, and 530 in the 5-year mortality risk, 5-year progression/relapse risk and time-to-relapse models for HR NB; and 522, 673, 604, 637, and 565 patients for the 1-year mortality risk, 2-year mortality risk, 1-year progression risk, time-to-death and time-to-relapse in the models for DIPG.

Regarding the variability of sample size as a function of the *R*^2^_Nagelkerke_, results show that including direct or indirect measures of the processes involved in the outcome to be predicted (*R*^2^_Nagelkerke_ = 0.5) as opposed to not doing so (*R*^2^_Nagelkerke_ = 0.15) strongly reduced the required sample size by an average of 71.2% (Table 4). This reduction in sample size was slightly lower when *R*^2^_Nagelkerke_ values of 0.5 and 0.8 were compared. Regarding the number of EPP variables, very high values (maximum 37.45) were found when the *R*^2^_Nagelkerke_ was 0.15, far above that of the classic 10 EPP. By contrast, when the R^2^_Nagelkerke_ was set to 0.8 the EPP value dropped to as low as 3.75.

**Table 4:** Variability of sample size with *R*^2^_Nagelkerke_

Model	***R***^**2**^_**Nagelkerke**_ = 0.15		***R***^**2**^_**Nagelkerke**_ = 0.5		***R***^**2**^_**Nagelkerke**_ = 0.8	
	Sample size	EPP	Sample size	EPP	Sample size	EPP
NB 5-year mortality risk	2383	24.39	666	6.82	550	5.63
NB relapse/progression risk	2495	21.42	704	6.04	590	5.06
NB time to death	2951	14.79	858	4.30	749	3.75
HR-NB 5-year mortality risk	2247	37.45	620	10.33	501	8.35
HR-NB 5-year relapse/progression risk	2280	31.01	631	8.58	513	6.98
HR-NB time to relapse/progression	2280	24.15	631	6.68	513	5.43
DIPG 1-year mortality risk	2247	33.70	620	9.30	501	7.52
DIPG 2-year mortality risk	2848	16.04	824	4.64	713	4.02
DIPG 1-year progression risk	2575	20.17	731	5.73	618	4.84
DIPG time to death	2705	16.72	775	4.79	663	4.10
DIPG time to progression	2419	20.69	679	5.81	563	4.82

Sample size calculations with different values of *R*^2^_Nagelkerke_ (0.15, 0.5, and 0.8). The number of events per predictor variable was obtained with the *pmsampsize* package

**Fig. 3:**
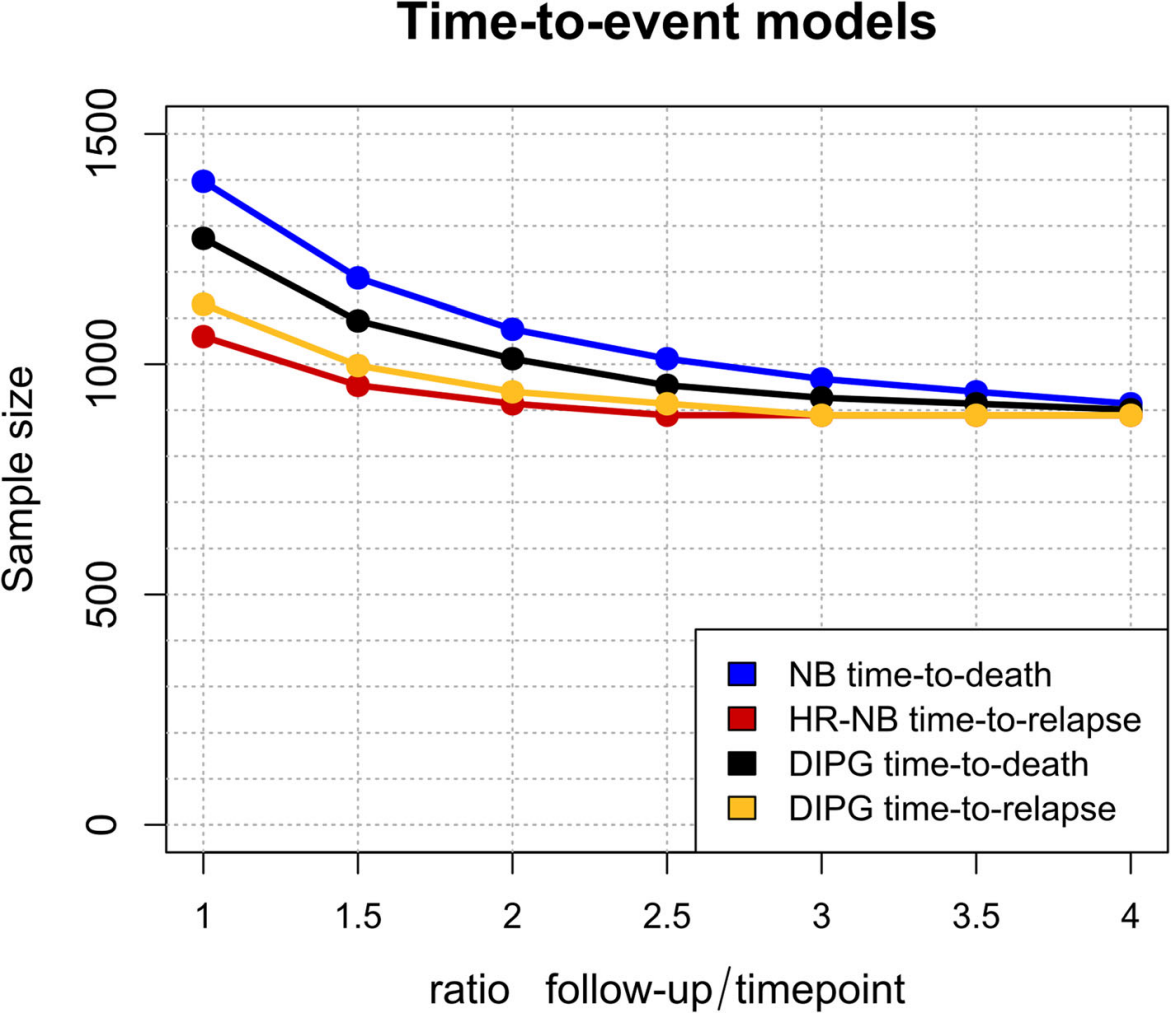
How the follow-up/timepoint ratio affects the sample size. Analysis of the variation in sample size for the time-to-event models relative to the ratio between the expected average follow-up of the dataset and the time points of interest for the predictions. *DIPG* diffuse intrinsic pontine glioma, *HR* high-risk, *NB* neuroblastoma

In addition, for time to event models, increasing the ratio between the time of follow-up and the time point of interest also leads to a lower sample size, with a reduction of between 13.8 and 23% when comparing a ratio of 1 and 2 (Fig. 3), although this reduction diminished as the ratio increased.

### Sample size requirements for model validation

Finally, the minimum sample size required to validate the predictive models, considered as 100 events, was 326 patients for the NB models, 246 for the HR NB models, and 592 for the DIPG models, with a desirable size (200 events) of 652, 491 and 1184 patients, respectively (Table 5).

**Table 5:** Sample sizes for external validation

Model	100 events	200 events
NB 5-year mortality risk	326	652
NB relapse/progression risk	288	776
NB time to death	326	652
HR-NB 5-year mortality risk	200	400
HR-NB 5-year relapse/progression risk	246	491
HR-NB time to relapse/progression	179	357
DIPG 1-year mortality risk	223	445
DIPG 2-year mortality risk	592	1184
DIPG 1-year progression risk	426	852
DIPG time to death	592	1184
DIPG time to progression	426	852

The sample size for the external validation of the models has been calculated considering a minimum effective sample size of 100 events and a desirable situation of 200 events

## Discussion

We have explored a practical solution to estimate the sample size necessary to develop robust clinical predictive models [13] to the specific case of the observational PRIMAGE project [5]. Unlike other estimation methods, such as the 10 EPP rule [7–9], this solution provides a set of algorithms to calculate the sample size required to construct and validate robust parametric predictive models based on model quality criteria, and type of clinical outcome.

The sample size obtained with the proposed methodology was compared to its analogous estimation predicted with other more basic rules, having significant discrepancies. In addition, when comparing the number of EPPs obtained when using this method with respect to the 10 and 5 EPP rules, the number of EPPs rise above 17 in some scenarios (17.38 EPPs for the HR-NB 5-year mortality risk model) but fall to as low as to 7 in others (NB 5-year mortality risk). This confirms that 10 and 5 EPP rules may not be generally applicable since the number of EPPs for sample size estimations might depend on the context of the study, the prevalence of the outcome, the quality of the predictor variables chosen and the type of model to be developed [10, 11].

To explore possible solutions for cases where the required sample size exceeds the available sample, the size variation relative to certain parameters used in the calculations was analysed (e.g. the number of predictor variables, estimated *R*^2^_Nagelkerke_ and follow-up/time point ratio). The most feasible option to decrease the required sample size is to reduce the number of predictor variables included in the model as the number of predictors and the sample size have a directly proportional relationship in the range of 5 to 30 predictor variables. One proposed strategy is to decrease the potential predictive variables to be included in the models. For this purpose, we propose to carry out an exhaustive manual selection process of the variables to be collected in the design phase of the study. To this end, it is of great important to have the opinion of experts in the field of interest, as well as to carry out a rigorous analysis of the related literature, thus selecting the candidate predictor variables considered most important in function of the outcome to be predicted [30, 31].

Other possible approaches are to include measures of the outcome to predict which would result in an increase of the *R*^2^_Nagelkerke_ value from 0.3 to 0.5, reducing the sample size by an average of 39.8 ± 0.7% (mean ± standard deviation).

Other mitigation strategies may include subject-wise cross-validation [32], resampling techniques [33], or data augmentation methods for medical images [34], and even exploring data imputation solutions for clinical data as suggested by Pezoulas et al. [35]. In some cases, the required sample size is not achievable even after applying sample reduction strategies. This is a study limitation, and researchers should be careful with the degree of evidence of the results.

Considering that the expected sample size in the PRIMAGE project is more than 2900 NB cases, of which at least 1500 are HR NB, the expected cohort is therefore appropriate to develop reliable models with up to 30 predictive variables. However, the number of DIPG patients expected in PRIMAGE project (*n* = 700) falls below the sample size estimated with the default parameters for the *pmsampsize* equations (1345 cases). A downsizing strategy should be considered by applying feature reduction/selection methods and reducing the number of predictive parameters. In this way, 673 DIPG cases would be required when the number of predictive variables included in the prediction models is set to 15.

It should also be highlighted that the most important step in clinical prediction models is the validation phase, in which the true fit of the model and its applicability to daily clinical practice is assessed to ensure reproducibility. Using the lower limit of 100 events, the minimum sample size obtained for the external validation of the PRIMAGE models was 326 patients for NB and 592 for DIPG, which is an achievable number in the case of NB but somewhat more challenging for DIPG given its lower incidence.

Regarding the possible biases, the *pmsampsize* formulas were developed considering only linear regression models for continuous outcomes, logistic regression for binary outcomes, or proportional hazards regression models for time-to-event data. These three different algorithms are parametric, suitable to obtain predictive models when the relationships between the different variables in the dataset are known and well-defined. However, when the relationships between variables are not direct, it seems more appropriate to apply non-parametric models that can efficiently exploit the more complex relationships between the variables, such as the k-nearest neighbours, support vector machines or decision tree algorithms. Therefore, the quality of the variables, the selection of the most appropriate algorithm for the data model, and the process of hyperparameter tuning are essential to obtain robust predictive models.

In summary, we have applied a recently devised method to determine sample sizes for clinical predictive model development and validation to the use-case of the observational PRIMAGE project, providing an overview of different sample size reduction approaches. This methodology is based on the epidemiological data and the nature of the outcome, tailoring the obtained sample size to the specific medical problem of interest. A common research framework for sample size estimation methodologies for the development and validation of clinical predictive models should be defined by the clinical research community.

## Data Availability

Not applicable

## Abbreviations

DIPG: Diffuse intrinsic pontine glioma
EPP: Events per predictor
HR: High-risk
NB: Neuroblastoma
OS: Overall survival
PFS: Progression-free survival
